# Research Progress of Plasma Cell Mastitis

**DOI:** 10.1002/iid3.70199

**Published:** 2025-04-27

**Authors:** Zhebin Liang, Lifeng Zhang

**Affiliations:** ^1^ Department of General Surgery The Fourth Affiliated Hospital of Soochow University Suzhou Jiangsu China

**Keywords:** granulomatous mastitis, IL‐6/JAK2/STAT3 pathway, mammary duct ectasia, plasma cell mastitis

## Abstract

**Background:**

Plasma cell mastitis (PCM), also termed mammary duct ectasia, is a chronic nonbacterial inflammatory disease characterized by mammary duct dilation and plasma cell infiltration. Due to its varied and nonspecific clinical presentation, PCM is frequently misdiagnosed as breast cancer, complicating clinical management.

**Objectives:**

This review aims to summarize recent advances in the understanding of PCM, focusing on its etiology, clinical manifestations, diagnosis, and treatment strategies, as well as clarifying differential diagnostic points with granulomatous mastitis (GLM).

**Methods:**

We reviewed recent literature highlighting clinical characteristics, diagnostic approaches, and therapeutic options related to PCM, including comparative studies addressing differences between PCM and GLM.

**Discussion:**

Recent progress has enhanced understanding of PCM's clinical and pathological features, yet distinguishing PCM from GLM remains clinically challenging due to overlapping presentations. An integrated approach involving clinical evaluation, imaging modalities, and histopathological examination is recommended to improve diagnostic accuracy and clinical outcomes.

**Conclusion:**

Further investigation into the pathogenesis of PCM is essential for developing more precise diagnostic criteria and effective treatments, ultimately improving patient prognosis and reducing misdiagnosis.

## 1 Introduction

Plasma cell mastitis (PCM), also called mammary duct ectasia, obstructing mastitis and comedo mastitis, was described for the first time by Burkitt in 1850 [1]. Formerly, it is assumed that PCM tends to occur in middle‐aged women. In 1974, Tedeschi and McCarthy reported the first case of PCM in men [2]. The incidence of PCM is difficult to calculate because many patients are not accurately diagnosed. According to rough estimates, the incidence of PCM is 2/10^5^, which is 1/20 of the incidence of breast cancer [3, 4]. PCM is characterized by a periductal phlogistic reaction, accompanied by ductal ectasia. The infiltration of abundant plasma cells and lymphocytes is the hallmark of this disease [5, 6]. The mechanism of PCM could be hyperprolactinemia or activation of IL‐6/JAK2/STAT3 signaling pathway [5, 7]. The clinical manifestations of PCM include noncyclical mastalgia, nipple discharge, nipple retraction, subareolar breast masses, and possible mammillary fistulae. These symptoms, which are similar to breast cancer, make it difficult to diagnose PCM. Surgical resection is considered in cases of complicated or recurrent PCM, but it is not typically the first‐line treatment unless complications such as abscess formation or persistent fistulas arise. Nonetheless, the recurrence rate after PCM resection is quite high, making PCM a major problem for surgeons [8, 9].

In recent years, many studies have confused PCM with granulomatous mastitis (GLM). In fact, they are not the same disease, although the clinical symptoms and treatment are similar [10, 11, 12]. Therefore, a clear distinction between PCM and GLM may be of great help in the treatment of PCM.

In summary, there are still many unresolved research questions regarding PCM, especially the pathogenesis of PCM and the differential diagnosis of PCM and GLM.

## 2 Pathogenesis of PCM

### 2.1 Prolactin

Studies from the 1980s suggested a possible link between prolactin (PRL) and PCM. Some reports indicate that nonpuerperal mastitis may be an early symptom of hyperprolactinemia, while others suggest that PCM itself could induce transient hyperprolactinemia (neurogenic hyperprolactinemia), lasting ~3 weeks [7, 13]. The proposed physiological mechanism involves thoracic wall nerve stimulation rather than direct breast injury or herpes zoster‐induced PRL elevation [14, 15]. PRL may also be involved in the regulation of immune and inflammatory responses [16]. Additionally, bromocriptine, a dopamine agonist that lowers PRL levels, has been reported to reduce recurrence in nonpuerperal mastitis [17]. The mechanism may be related to the inhibitory effect of bromocriptine on PRL [18, 19]. However, more recent evidence is needed to confirm this effect in PCM specifically.

### 2.2 IL‐6/JAK2/STAT3 Pathway

In recent studies, researchers have demonstrated that activation of IL‐6/JAK2/STAT3 signaling pathway induces PCM in mice [4]. According to the current research results, it was found that IL‐6 is not only involved in immune response, but also in inflammation, hematopoiesis, bone metabolism, and embryonic development [20, 21, 22]. It has been known from relevant studies: (1) IL‐6 level was significantly higher in PCM patients than in acute mastitis patients or normal group. (2) Noticeable higher IL‐6 expression was found in PCM patients with nipple retraction than in other PCM patients. (3) Bcl‐2 level was higher in PCM patients than in acute mastitis patients or normal group, but there was no difference in Bcl‐2 immunostaining between PCM patients experiencing recurrence and other PCM patients [4, 5].

### 2.3 Other Mechanisms

Although PCM patients with nipple retraction exhibit higher IL‐6 expression, the underlying cause of this elevation remains unclear. Additionally, Bcl‐2 plays a role in PCM pathogenesis by inhibiting plasma cell apoptosis and regulating lymphocyte survival in a mitochondria‐dependent manner. The possible physiological mechanisms are described below: the transcription factors, for example, B lymphocyte induced maturation protein 1 (Blimp‐1) which plays a number of crucial roles in the adaptive immune system and X‐box binding protein 1 (XBP‐1), are both vital for the differentiation of plasma cells [23, 24]. Blimp‐1 induces the transcription of XBP‐1 and promotes plasma cell differentiation. IL‐6/STAT3 pathway works precisely by inducing Blimp‐1 expression through the activation of STAT3, and simultaneously, XBP‐1 increases the production of IL‐6, forming a positive feed‐forward loop. In ductal epithelium cell, IL‐6/STAT3 pathway activation can result in increased expression of intercellular adhesion molecule‐1 (ICAM‐1). ICAM‐1 is overexpressed in ductal epithelium in PCM and promotes infiltrating inflammatory cell homing to the ductal epithelium, which can lead to obvious degenerative changes, while ICAM‐2 and E‐selectin in ductal epithelium were no significant differences between PCM patients and normal controls [5, 25]. Although PCM patients with nipple retraction have the higher IL‐6 expression, we still do not know exactly what causes the high expression of IL‐6. In addition, Bcl‐2 can inhibit plasma cell apoptosis by regulating the survival of lymphocyte in a mitochondria‐dependent way [5].

In addition to the above, there are other possible causes of PCM, such as smoking, anaerobic infection, autoimmune causes, overweight/obesity, and the late onset of menarche [26].

This has been known for a long time that smoking is a major factor not only in the etiology of periductal mastitis but also in the etiology of duct ectasia. However, these researchers have come up with a new idea. They consider that smoking can only cause periductal mastitis, not duct ectasia [27, 28].

To sum up, most of the current studies on the pathogenesis of PCM are still in the stage of animal experiments, and there are few studies with real clinical significance. In view of the current situation, it is very important to translate the research results into the clinic as soon as possible.

## 3 Clinical Manifestations and Stages of PCM

### 3.1 Clinical Manifestations of PCM [29, 30]

1. Palpable hard lumps with poorly defined boundaries and limited mobility.
2. Redness, swelling, warmth, and pain in or around the areola, sometimes accompanied by a pulsating sensation.
3. Nipple retraction (observed in a small percentage of cases), nipple discharge (present in nearly half of cases, which may be profuse and vary in color from green to yellow to brown), and fistula formation. Associated abscesses tend to be indolent.

### 3.2 Clinical Stages of PCM

PCM can be divided into four disease stages, namely, duct dilatation, inflammation, abscess, and fistula. These four disease stages have different etiologies and pathogenesis (Table 1).

**Table 1:** Clinical stages of PCM.

Stage	Features
Duct dilatation stage	Noncyclical mastalgia, nipple discharge, nipple retraction, and subareolar breast lump
Inflammation stage	Skin redness, swelling, heat pain, and skin lesions
Abscess stage	Abscess formation
Fistula stage	Fistula and abscess ruptured

#### 3.2.1 Duct Dilatation Stage

As the initial stage of the disease course of PCM, the duct dilatation stage is often devoid of inflammatory signs. The main clinical manifestation of this stage is noncyclical mastalgia (the pain is usually in the nipple and areola), nipple discharge (some patients may develop milk‐like discharge when the nipple is squeezed), nipple retraction, a subareolar breast lump, etc. [30]. The main imaging manifestations at this stage are as follows:

1. Ultrasound (US) examination: The lesions are mostly flat, with sparse blood flow, low flow rate, and little resistance. The most common US findings of ductal ectasia are dilated ducts and tubular anechoic lesions under the areola that may contain hyperechoic debris [31, 32].
2. Mammography examination: The lesions show asymmetric shadows of increased density, flame‐like appearance, uneven density, low‐density tubular structure, and scattered rod or hollow small round calcification along the long axis of the mammary duct.
3. Multislice helical CT examination: The lesions show small round nodules around the lesion, thickened skin in the areola area, and widened soft tissue shadow in the main mammary duct.
4. Transmission electron microscopic features of duct dilatation stage were denudation of the epithelial cells with focal loss of microvilli, widening of the interepithelial junctions with focal disruption of the T bars, periductal collagenization without inflammation, and features suggestive of epithelial mesenchymal transition (EMT) [33].

As for the pathogenesis of ductal dilation, there are several main views as follows:

1. Autoimmune disease (AID): There are fewer CD4^+^CD25^+^ regulatory T cells in peripheral blood of PCM patients, and the levels of transforming growth factor‐β (TGF‐β) and forkhead box P3 (FOXP3) transcription are lower [34].
2. Hormone fluctuation: Nonpuerperal mastitis may induce transient hyperprolactinemia (neurogenic hyperprolactinemia) of about 3 weeks' duration [7, 13, 35].
3. Abnormal breast structure: Inverted or malformed nipples, ductal stenosis, and breast dysplasia can result in occlusion of the mastoid foramen, obstruction of metabolite excretion, or ductal dilatation [8, 36].
4. Other views: Smoking, age, drugs, etc. [27, 28, 37]. Along the long axis of the catheter.

#### 3.2.2 Inflammation Stage

The inflammatory stage often follows the duct dilatation stage, but some patients skip the duct dilatation stage and directly enter the inflammatory stage. The main clinical symptoms of this stage are skin redness, swelling, heat pain, and skin lesions gradually increased.

The main pathogenesis of the inflammatory stage is cytokines. Because ICAM‐1 mediates leukocyte adhesion, the overexpression of ICAM‐1 in PCM in ductal epithelium promotes the homing of infiltrating inflammatory cells to ductal epithelium, which can lead to obvious degenerative changes [5, 25, 38, 39].

#### 3.2.3 Abscess Stage

After the inflammatory stage, PCM often enters the abscess stage as the disease course progresses without treatment.

The abscess stage is mostly caused by the following reasons: (1) Bacteria: Over the last century, *Enterococcus*, anaerobes, *Staphylococcus aureus*, and *Bacteroides* have been isolated and successfully cultured from nipple secretions of patients with PCM [40]. There are also some ideas that PCM is associated with nontuberculous mycobacterial infections [29, 41]. (2) IL‐6: IL‐6 promotes the activation of JAK/STAT signaling pathway by binding to glycoprotein 130, and then upregulates the expression of antiapoptotic genes Bcl‐2 and ICAM‐1. Bcl‐2 can prolong the survival time of plasma cells, and ICAM‐1 can promote the penetration of plasma cells into the ductal epithelium. Activation of this signaling pathway also stimulates the differentiation of B cells into plasma cells, effectively forming a positive feedback loop for IL‐6/JAK/STAT signaling [5, 20, 21, 22, 25]. All these factors contribute to the progression of abscess.

#### 3.2.4 Fistula Stage

The fistula stage is not the last stage of PCM, but the stage with the worst prognosis of PCM. This is mainly because of the higher recurrence rate of fistula treatment [42]. The abscess ruptured and emerged, resulting in difficulty in healing the fistula.

The course of PCM can be divided into the duct dilatation stage, inflammation stage, abscess stage, and fistula stage. A duct dilatation stage is characteristic. However, these four stages do not necessarily occur sequentially. Prompt therapeutic intervention can also prevent further progression of the disease.

## 4 Diagnosis and Differential Diagnosis of PCM

### 4.1 Diagnosis of PCM

The main points of the current clinical experimental diagnostic criteria for PCM are as follows:

1. Nonlactating and nonpregnant women;
2. With the clinical manifestation as described above;
3. Fine needle puncture biopsy showing abundant infiltrated plasma cells without malignant tumor cells; and
4. Auxiliary examinations, such as breast US, magnetic resonance imaging (MRI), and computed tomography (CT), are consistent with clinical manifestations [30, 43, 44, 45].

#### 4.1.1 Imaging Diagnosis of PCM

Among the above points, imaging diagnosis is undoubtedly the most important. It is well known that the difficult point of imaging diagnosis of PCM is the differentiation from breast cancer [29, 31, 46, 47, 48]. From some recent articles, we found some recent advances in the imaging diagnosis of PCM. Researchers found that all the PCM and breast cancer patients presented hyperenhancement of contrast‐enhanced ultrasound (CEUS) breast lesions compared with the normal control group. The difference between PCM and breast cancer is that most of PCM exhibited perfusion defect of CEUS patterns with smooth edge and multiple lesions, while only a minority of breast cancer patients showed perfusion defect [32]. The possible reason is that breast lesions of PCM are completely enclosed focal pus cavities full of secretions which caused by intense chronic inflammation [42, 49, 50]. In addition to CEUS, superb microvascular imaging (SMI) also has a certain diagnostic value. In related studies, the majority of breast cancer patients displayed different degrees of calcification, while most PCM were in regular shape and shown no calcification. In regard to blood flow parameters, PCM obtained significantly lower mean value of resistance index (RI) and pulsatility index (PI) compared with malignant lesions. The sensitivity, specificity, and accuracy rate of US plus SMI was observably greater than US alone [51].

#### 4.1.2 Blood Cell Analysis of PCM

Except imaging diagnosis, blood cell analysis cannot be ignored either. White blood cell count (WBC), neutrophils, and neutrophils/lymphocytes ratio of blood cell analysis in PCM patients increased remarkably compared with other mastitis patients [32].

### 4.2 Differential Diagnosis of PCM

There are many diseases that should be distinguished from PCM, such as breast cancer and other mastitis. The differential diagnosis between PCM and breast cancer has become easier with the development of modern auxiliary diagnostic techniques, such as the abovementioned CEUS. So the differential diagnosis between GLM and PCM is mainly discussed here (Table 2).

**Table 2:** The differential diagnosis between PCM and GLM.

	Plasma cell mastitis	Granulomatous mastitis	
Pathogenesis	Hyperprolactinemia		
	Smoking		
	Autoimmunity		
	The activation of IL‐6/JAK2/STAT3 pathway	α1‐antitrypsin deficiency	
	Anaerobic infection	Oral contraceptives	
		Gestation, birth, and breastfeeding	
	Overweight/obesity	Microbiological agents	
	The late onset of menarche	Ethnicity	
Histological features (infiltrated cells)	Plasma cells		
	Lymphocytes		
		Epithelioid histiocytes	
		Multinucleated giant cells	
		Eosinophils	
		Neutrophils	
Past medical history	Nonlactating and nonpregnant women	80% of patients developing GLM within 4 years after weaning	
Clinical manifestations	Hard lumps (indistinctive boundaries and poor mobility)		
	Redness, swelling, heat, and pain		
	Nipple retraction, nipple discharge, and fistula formation	Only nipple retraction	
Diagnostic criteria	Nonlactating and nonpregnant women		
	With the clinical manifestation as described above		
	Fine needle puncture biopsy showing abundant infiltrated plasma cells without malignant tumor cells		Fine needle puncture biopsy showing abundant infiltrated epithelioid histiocytes and multinucleated giant cells without malignant tumor cells
	Auxiliary examinations consistent with clinical manifestations		

In terms of pathogenesis, the same etiology of GLM and PCM is as follows: hyperprolactinemia, smoking, and autoimmunity. As for the differences, the potential pathogenesis of GLM is as follows: α1‐antitrypsin deficiency, oral contraceptives, microbiological agents, ethnicity, gestation, birth, and breastfeeding, while the pathogenesis of PCM is these points: the activation of IL‐6/JAK2/STAT3 pathway, anaerobic infection, overweight/obesity, and the late onset of menarche [52, 53, 54].

Histologically, GLM is characterized by the infiltration of epithelioid histiocytes, multinucleated giant cells, plasma cells, lymphocytes, eosinophils, and neutrophils, while the hallmark of PCM is the infiltration of abundant plasma cells and lymphocytes without epithelioid histiocytes and multinucleated giant cells [55, 56, 57].

In addition, a key clinical distinction is that almost all GLM patients have breastfeeding history, with 80% developing the disease within 4 years after weaning. In contrast, PCM typically begins in the retroareolar region and remains confined to this area, whereas GLM often starts peripherally and may extend to the retroareolar region. Furthermore, approximately half of GLM patients report breastfeeding difficulties, which is a crucial feature distinguishing it from PCM [58].

The clinical manifestations and diagnostic criteria of GLM and PCM are similar. The only difference, which remains to be proven, is that GLM does not seem to cause nipple discharge [53, 54].

The diagnosis of PCM differs from GLM in pathogenesis and histologic features. Both have their unique pathogenesis. In addition, GLM showed more types of inflammatory cell infiltration than PCM. These differences suggest that PCM and GLM are not the same disease, so it is important to study their pathogenesis and take targeted treatment measures.

## 5 Therapy of PCM

Up to now, surgical excision is the major treatment of PCM [8, 25, 49]. Although surgical excision has become the mainstream treatment method, the high postoperative recurrence rate cannot be ignored [8, 9]. In addition, there is no specific drug to treat PCM, so it is very important to find new treatment methods [8, 49, 59].

### 5.1 Surgical Treatment

In recent years, many studies have put forward new views on the surgical treatment of PCM: (1) for deep fistula caused by periductal mastitis, total resection of catheter system combined with fistula should be performed under antibiotic treatment to ensure the lowest recurrence rate [60]. (2) Continuous postoperative negative pressure irrigation assisted mammaplasty may have effect in the treatment chronic refractory PCM [42].

### 5.2 Ultrasound‐Guided Microwave Ablation

In recent years, some doctors have applied microwave ablation technology to the treatment of PCM. In the past, this technique was often used to treat benign breast tumors. The report showed that the effective rate of the ablation group was much higher than that of the surgery group (86.8% vs. 46.7%), and the time to complete disappearance of the lesion in the ablation group was shorter than that in the surgery group (75.55d vs. 103.87d). This indicates the prospect of microwave ablation in the treatment of PCM. However, we remain skeptical about this technique because of the small number of cases reported (38 in the ablation group and 30 in the surgery group) [61]. It is hoped that there will be further research in this direction in the future to confirm the effectiveness of microwave ablation technology.

### 5.3 Drug Treatment

#### 5.3.1 Hormone Therapy

In fact, surgical excision is not the only treatment method. Hormone therapy has been widely used in clinic. Including estrogen suppression therapy and the use of glucocorticoids. However, they have not been very effective, and many patients relapse after stopping the drug. This may be due to the fact that PCM is an AID and glucocorticoids only temporarily suppress the immune response and do not cure the disease.

#### 5.3.2 IL‐6/JAK2/STAT3 Pathway Inhibitor

Actually, surgical excision is not the only treatment. It has been mentioned above that activation of the IL‐6/JAK2/STAT3 pathway induces PCM in mice. In theory, the medicine which can inhibit the progression of PCM mainly through downregulating IL‐6/JAK2/STAT3 levels may be effective in the treatment of PCM [4, 5, 49].

#### 5.3.3 Sinomenine Hydrochloride (SH) and Alkaloid Sinomenine (SN)

Numerous studies have demonstrated that SH has potent anti‐inflammatory and immunoregulatory properties [49, 62, 63, 64, 65, 66, 67]. This suggests that SH may play a role in the treatment of PCM. From some of the past literature, we can learn the following information. The SN, an active compound isolated from the Chinese medicinal plant Sinomenium acutum, has been successfully used to treat various AIDs in Chinese traditional medicine for centuries [62]. Previous studies have shown that SN has a wide range of effects, including anti‐inflammation, immunosuppression, arthritis amelioration, and preventing LPS‐induced hepatitis [62, 68]. SN also inhibited splenocyte proliferation and B cell production of antibodies, and effectively reduced macrophage production of inflammatory factors, including TNF‐α, IL‐1, and nitric oxide (NO) [62]. In addition, the combination of SN and suboptimal dose of cyclosporine A (CsA) has a synergistic effect, which can suppress the immune response and prolong the survival time of rat heart transplantation [63].

In recent years, researchers have found evidence that SH may have a potential therapeutic effect for PCM by means of IL‐6/JAK2/STAT3 signaling pathway [49]. In the first place, researchers found that SH inhibited phosphorylation of STAT3 [69]. On this basis, SH was confirmed to inhibit PCM progression mainly by downregulating IL‐6/JAK2/STAT3 levels [49]. These studies provide support for the clinical application of SH in the treatment of PCM in theory.

#### 5.3.4 MCC950

MCC950 is an NOD‐like receptor family, pyrin domain containing‐3 protein (NLRP3) inhibitor. It can inhibit plasma cell invasion by increasing the number and enhancing the function of myeloid‐derived suppressor cells (MDSC), which may be used to treat PCM [70].

In view of the current treatment options, surgical excision is traditional with limited efficacy and high recurrence rate. Microwave ablation can achieve good results, but more clinical data are needed to verify it. Drug treatments targeting the pathogenesis have shown promising results in animal trials, but further clinical trials are needed. Based on the clinical stage of PCM, it is clear that prompt treatment can significantly improve the prognosis and reduce the risk of recurrence.

## 6 Discussion

PCM is a relatively rare form of mastitis characterized by breast ductal dilatation and plasma cell infiltration. Its clinical manifestations are similar to those of breast cancer and other mastitis, which makes its diagnosis and treatment somewhat difficult. Through reviewing the studies over the years, we found that the diagnosis of PCM has become easier with the development of assistive techniques. Therefore, the etiology and treatment of PCM have become difficult. Although many studies on the etiology of PCM have been published in recent years, few have made substantial progress. In addition to the traditional surgical treatment, we believe that microwave ablation technology has certain application value. In terms of medication, we believe that inhibition of the IL‐6/JAK2/STAT3 signaling pathway is a viable research direction, and related inhibitors have the potential to be used in the treatment of PCM to assist the current surgical treatment with high recurrence rate. NLRP3 inhibitors (such as MCC950) are also potential future drug treatments. In addition, it may be of great value to study the pathogenesis of PCM caused by hyperprolactinemia. However, we have not found any relevant studies on the use of PRL in the treatment of PCM, which may be the direction of future research. In addition to the study of the pathogenesis of PCM, it is also important to study the pathogenesis of GLM. Because GLM and PCM are often treated as the same disease, with the same treatment. This may also be one of the reasons for the current high recurrence rate of surgical resection.

## 7 Conclusions

PCM is a challenging disease from diagnosis to treatment. Although many articles have been published on the diagnosis and treatment of PCM, these studies have not yet had an impact on clinical practice. In addition, there are still many scholars who confuse GLM and PCM, which further obscure our understanding of PCM. Therefore, we should further study the pathogenesis of PCM to solve this intractable problem from the source.

## Author Contributions

**Zhebin Liang:** data curation, formal analysis, writing – original draft, writing – review and editing. **Lifeng Zhang:** data curation, project administration, supervision, writing – original draft, writing – review and editing.

## Supporting information

PRISMA 2020 checklist.

## Data Availability

The authors have nothing to report.
